# Substance Use-Associated Mortality among Heart Donors after the COVID-19 National Emergency Increased but Did Not Affect Peri-Transplant Outcomes

**DOI:** 10.3390/jcdd10050222

**Published:** 2023-05-20

**Authors:** Meg Fraser, Bellony Nzemenoh, Scott Jackson, Thanat Chaikijurajai, Robert Halmosi, Kalman Toth, Wahab J. Khan, Tamas Alexy

**Affiliations:** 1 Department of Medicine, Division of Cardiology, University of Minnesota, Minneapolis, MN 55455, USA; mfraser10@umphysicians.umn.edu; 2 Department of Medicine, University of Minnesota, Minneapolis, MN 55455, USA; nzeme001@umn.edu (B.N.); chaik013@umn.edu (T.C.); 3 Analytics Consulting Services, MHealth Fairview, Minneapolis, MN 55455, USA; scott.jackson@fairview.org; 4 Division of Cardiology, 1st Department of Medicine, Medical School, University of Pecs, 7624 Pecs, Hungary; halmosi.robert@pte.hu (R.H.); toth.kalman@pte.hu (K.T.); 5 Department of Medicine, Avera Health, Sioux Falls, SD 57105, USA; wahab.khan@usd.edu

**Keywords:** heart transplant, transplant outcomes, donor characteristics, COVID-19 pandemic, mental health disorders, substance use disorder

## Abstract

Introduction: The COVID-19 pandemic and consequent social isolation prompted a surge in mental health disorders and substance use in the general population and, therefore, in potential organ donors. We aimed to evaluate if this led to a change in donor characteristics, including the mechanism and circumstance of death, and how this may have affected clinical outcomes following heart transplantation. Methods: We identified all heart donors from the SRTR database between 18 October 2018 and 31 December 2021, excluding those who donated immediately after the US national emergency declaration. Donors were stratified into pre-COVID-19 (Pre-Cov; through 12 March 2020) and post-COVID-19 national emergency declaration cohorts (Post-Cov; 1 August 2020 through 31 December 2021) based on the heart procurement date. Relevant demographics, cause of death, and substance use history were collected in addition to graft cold ischemic time, the incidence of primary graft dysfunction (PGD), and recipient survival at 30 days post-transplant. Results: A total of 10,314 heart donors were identified; 4941 were stratified into the Pre-Cov and 5373 into the Post-Cov cohorts. There was no difference in demographics, but illicit drug use was significantly higher in the Post-Cov group, leading to an increased incidence of death from drug intoxication. Fatal gunshot wounds were also more common. Despite these changes, the incidence of PGD remained similar (*p* = 0.371), and there was no difference in 30 days recipient survival (*p* = 0.545). Conclusion: Our findings confirm that COVID-19 had a major impact on mental health and psychosocial life with an associated increase in illicit substance use and fatal intoxication rates in heart transplant donors. These changes did not alter peri-operative mortality following heart transplantation. Future studies are needed to ensure that long-term outcomes remain unaffected.

## 1. Introduction

The Severe Acute Respiratory Syndrome Coronavirus-2 (SARS-CoV-2) was first identified in China in December 2019 and spread rapidly around the world, prompting the coronavirus disease-19 (COVID-19) pandemic. In an attempt to limit viral transmission as much as possible, most local governments instituted business closures, mandating the transition of non-essential services to virtual platforms as feasible, as well as the immediate cancellation of social and recreational events. In addition, restrictive laws were introduced prohibiting travel, especially international, and gatherings outside of the immediate family. Similarly, the United States (US) administration enacted a national emergency (NE) on 13 March 2020, forcing businesses to shut down across the country, thus leading to widespread workforce layoffs and early retirements. These necessary public health regulations further enforced social distancing and prompted a sense of isolation, adding to the significant strain already encountered by the population. Common stressors during the early phases of the pandemic included fear of contracting the virus without specific treatment options, death of close friends and loved ones, restricted hospital staff and bed availability to provide the highly specialized care necessary, inaccurate, or contradicting information conveyed to the public by officials, markedly limited availability of daily consumables, the feeling of uncertainty, and the mounting financial strain due to income loss in the setting of rising expenses. The combination of these factors led to a surge in mental health disorders (MHD) and substance use disorders (SUD) on top of the already widespread opioid epidemic in the US.

The COVID-19 pandemic had a profound effect on SUD in many ways. Firstly, it led to an increase in illicit drug use related to the mounting stress and social isolation. Secondly, many in-person addiction treatment center and program activities were disrupted during the early phase of the pandemic which, undoubtedly, affected the ability to seek much-needed support. Finally, a less openly discussed effect was the major disruption in the supply chain that not only affected common goods, but also select illicit substances. This prompted the use of increasingly potent drugs, most certainly contributing to the rising number of fatal overdoses during and after the NE, especially among the younger generation [1,2]. In the US, commonly used illicit drugs include prescription opioids, cocaine, methamphetamine, and heroin. Cannabis addiction is widespread, although it is currently legalized in several states. According to the National Survey on Drug Use and Health from 2020, 5.3% of the US population over the age of 12 met the criteria for alcohol use disorder, which is defined as a pattern of alcohol use that leads to clinically significant impairment or distress, based on two or more (of 11) possible symptoms within a 12-month period [3]. These behavioral and epidemiological shifts are of critical importance for the transplant community as organs, including the heart, are donated by the population segment most frequently affected by SUD.

Treatment options for symptomatic, end-stage (Stage D) heart failure (HF) include medical therapy with continuous inotrope infusion (such as milrinone and dobutamine), durable left ventricular assist device (LVAD) implantation, and orthotopic heart transplantation (HT). While technological advancements have rendered LVAD therapy more lucrative in recent years, HT remains the most definitive treatment for HF that not only provides the best long-term clinical outcomes, but also the highest quality of life [4]. Although a very significant organ supply/demand mismatch remains, there has been a recent increase in donor heart availability and their utilization. This prompted the annual number of HT surgeries performed in the US to surpass 4000 for the first time in 2022 [5]. Contributing factors include: (1) the growing problem of the deadly opioid epidemic across the country; (2) increasing organ acceptance from hepatitis C antibody positive (prior infection that has been cured with the virus successfully eliminated) as well as antigen positive donors (those with active disease and detectable viral load in the blood); (3) the recent introduction of novel procurement methodologies such as donation after circulatory death (DCD) using the Transmedics Organ Care System (OCS; Transmedics Inc., Atlanta, GA, USA) and normothermic regional perfusion utilizing veno-arterial extracorporeal membrane oxygenation (VA-ECMO) technology; and (4) the development of novel organ transport systems such as the SherpaPak^®^ (Paragonix Technologies Inc., Cambridge, MA, USA). Nevertheless, as of today, grafts procured from brain-dead donors remain the primary organ source for cardiac transplantation. The majority of these donors suffer an irreversible, non-survivable brain injury as a result of severe trauma often caused by a violent accident, suicide, homicide, or due to cardiopulmonary arrest in the setting of an illicit drug overdose. The manner of donor death and, specifically, the duration of “low flow” time, when the organ is under-perfused due to a combination of severe hypotension and “catecholamine storm”, may significantly impact its viability, the incidence of primary graft dysfunction (PGD), functional recovery, and thus, recipient survival in the immediate post-transplant period and beyond. As detailed above, the profound psychosocial effects of the COVID-19 pandemic may have prompted a shift in donor characteristics, including a change in the most common cause, mechanism, and circumstance of death. We thought to evaluate such potential changes among heart transplant donors and their impact on the incidence of PGD as well as 30 days post-HT survival rates.

## 2. Methods

### 2.1. Data Source

This study used data from the scientific registry of transplant recipients (SRTR). The SRTR data system includes data on all donors, waitlisted candidates, and transplant recipients in the US, submitted by the members of the Organ Procurement and Transplantation Network (OPTN). The Health Resources and Services Administration (HRSA), US Department of Health and Human Services provides oversight to the activities of the OPTN and SRTR contractors. All authors were approved to participate in the current project by the SRTR.

### 2.2. Aims of the Study

The aims of the present study were to evaluate select changes in the US heart donor characteristics related to the COVID-19 pandemic and their potential effect on post-transplant clinical outcomes. Specifically, we sought to compare the prevalence of MHD and SUD history as well as the common causes, mechanisms, and circumstances of donor death in the eras before and after the US COVID-19 NE declaration. We also aimed to compare the potential change cold ischemic time and the proportion of organs procured by the local Organ Procurement Organization (OPO) versus non-local OPOs (indirect measure of organ travel and placement efficiency). Lastly, we contrasted the above findings with the incidence of PGD and recipient survival rates at 30 days post-cardiac transplantation. Our first hypothesis was that rates of illicit drug-related deaths would be increased in heart donors after the onset of the COVID-19 pandemic. Our second hypothesis was that there would be an increase in graft cold ischemic time and the proportion of organs procured by non-local OPOs in the post-COVID-19 NE declaration era (Post-Cov).

### 2.3. Study Population

The pre-COVID-19 pandemic period (Pre-Cov) was set between 18 October 2018 and 12 March 2020. The date 18 October 2018 was selected as it represents the date when the updated United Network of Organ Sharing (UNOS) heart allocation policy was implemented across the US. The primary aim of the new policy was to reduce waitlist mortality by prioritizing patients at the highest risk for death, such as those requiring a temporary mechanical circulatory support device to maintain adequate hemodynamics, to more acute urgency listing status. As intended and anticipated, it led to significant changes in candidate listing strategies across centers, pre-transplant management approaches, and organ distribution, potentially confounding our analysis if donors from both allocation eras were included. Therefore, this study was limited to the post-heart allocation change time period. The date 13 March 2020 is the date when the COVID-19 NE was declared in the US. As such, we set 12 March 2020 as the final day of the Pre-Cov period.

Post-Cov period was defined as 1 August 2020 through 31 December 2021. As SARS-CoV-2 continued to spread across the country in early 2020, hospitals were overwhelmed and understaffed, mandating them to focus primarily on caring for patients with the most severe COVID-19 disease. Consequently, transplant programs not only reduced their volumes to the minimum, but data reporting was also uncertain and often incomplete during the early phases of the pandemic. Therefore, we chose 1 August 2020 as the first day of the Post-Cov period. The date 31 December 2021 is the most recent date with appropriate follow-up data available to authors in the SRTR database at the time of this analysis.

We identified all heart donors from the SRTR database between 18 October 2018 and 31 December 2021 who were stratified into Pre-Cov and Post-Cov cohorts based on the organ procurement date as defined above. We collected relevant demographic information such as age, gender, and race distribution for these donors. The cause of death (anoxia, cerebrovascular accident/stroke, head trauma, CNS malignancy, other), the mechanism of death (cardiovascular causes, drug overdose, gunshot wound) as well as the circumstance (suicide vs. homicide) were recorded, in addition to the substance use history (cigarette smoking, heavy alcohol consumption, cocaine, and other illicit drug use), recipient urgency listing status at the time of transplant surgery, type of OPO, graft cold ischemic time, and the post-transplant incidence of PGD. Recipient survival rates at 30 days were compared between the Pre-Cov and Post-Cov cohorts using Kaplan–Meier analysis. Those with a pre-transplant temporary or durable mechanical circulatory support device were included, but multi-organ transplant recipients were excluded from the current analysis. Donors that met DCD criteria at the time of procurement were also excluded for multiple reasons: (1) not all centers across the US accept DCD donor offers; (2) DCD donation increased significantly after it was accepted as standard of care (incidence of 0.5% vs. 4.7% in the Pre- and Post-Cov periods, respectively); (3) grafts placed on OCS may be transported to the recipient from significantly longer distances, thus increasing reported graft ischemic time; and (4) the incidence of PGD may potentially be higher after DCD donation although a clear association is yet to be established. No transplantations using the NRP strategy were included in the SRTR data file used for the current analysis. Cold ischemic time refers to the time between aortic cross-clamp placement at the time of organ procurement and reperfusion of the graft in the recipient. PGD is defined as graft dysfunction within 24 h of transplant surgery.

### 2.4. Statistical Analysis

Baseline characteristics between the study cohorts were compared using chi-square or Fisher’s tests for categorical variables and t-tests for continuous variables. Data are presented as mean ± SD for continuous variables and number (percentage) for categorical variables. In addition, Kaplan–Meier analysis was performed to compare all-cause mortality between the Pre-Cov and Post-Cov recipient cohorts, censored at 30 days post-transplant. *p*-values less than 0.05 were considered statistically significant. Statistical analyses were performed using R version 4.2.1 (R Core Team. R: A language and environment for statistical computing. R Foundation for Statistical Computing, Vienna, Austria). Website: https://www.R-project.org/, accessed on 4 April 2023.

## 3. Results

A total of 10,037 heart donors were identified from the SRTR database within the pre-specified time frame who met the inclusion criteria and were included in the present analysis. Of these, 4915 were assigned to the Pre-Cov and 5122 to the Post-Cov cohort. Mean donor age, race, gender, history of substance use (heavy alcohol, tobacco, cocaine, other illicit drugs), as well as the mechanism of death (illicit drug overdose, gunshot wound, intracranial hemorrhage) and circumstance (homicide vs. suicide) are depicted in Table 1.

We found no significant difference in donor age (29.9 ± 13.2 vs. 30.1 ± 12.6 years; *p* = 0.54) and gender (69.5% vs. 70.3% male; *p* = 0.41) between the Pre-Cov and Post-Cov cohorts. Fewer organs were procured from white (78.3% vs. 80.2%) and more from African American donors (18.3% vs. 16.2%) in the Post-Cov era (*p* = 0.026). A significantly higher proportion of donors from the Post-Cov group had a history of any illicit drug use (83.4% vs. 80.9%; *p* < 0.001). However, when comparing cocaine specifically, which is the only drug listed separately in SRTR, its use was found to be less frequent in the Post-Cov era (23.3% vs. 25.1%; *p* = 0.036). The exact reason for this decline remains to be determined. As it is often used by adolescents, contributing factors may include difficulties obtaining/using the drug and disruptions in peer group gatherings that solicit use. However, the combination of these data may indirectly suggest that overdose from illicit substances other than cocaine, such as opioids and methamphetamine, have increased significantly during the pandemic. The prevalence of active smoking was essentially identical (10.7% vs. 10.7%; *p* = 0.98), and while there was a trend toward increased alcohol consumption in the Post-Cov cohort, the difference did not reach statistical significance (16.9% vs. 16.0%; *p* = 0.228).

Mirroring donor social history, death from drug intoxication was significantly higher in the Post-Cov cohort (23.3% vs. 19.5%; *p* < 0.001), as was the incidence of fatal gunshot wounds (16.9% vs. 15.1%; *p* = 0.01). The overwhelming majority of donors died from anoxia or head trauma in both eras (84.3% combined vs. 85.8% combined). There was a decrease in donor deaths deemed to be caused by cardiovascular causes in the Post-Cov group (8.5% vs. 9.8%; *p* = 0.04) with no clear explanation for this finding as of yet. However, it is consistent with papers reporting lower incidence of heart failure exacerbations or acute myocardial infarction during and immediately following the COVID-19 pandemic NE [6,7,8,9].

**Figure 1:**
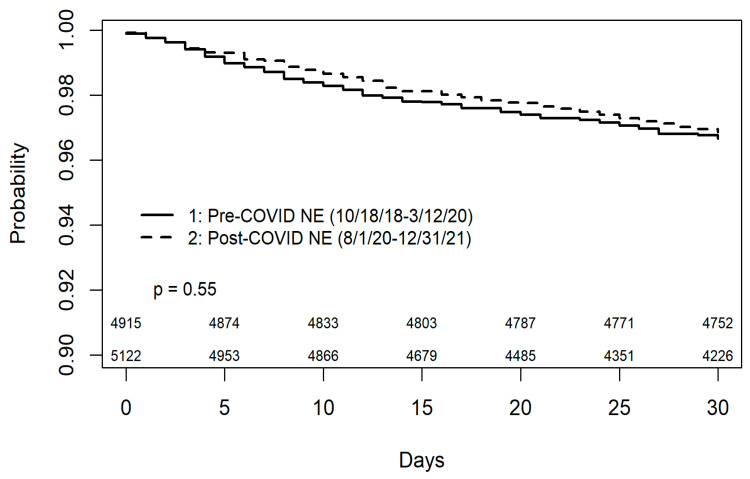
Kaplan–Meier analysis comparing 30-days recipient survival in the Pre-COVID-19 (Pre-COVID NE) and Post-COVID-19 national emergency declaration (Post-COVID NE) cohorts. Recipient survival rates were high in both groups, exceeding 96% with the difference not reaching statistical significance (*p* = 0.55). The Pre-COVID-NE era was defined as the period between 18 October 2018 and 12 March 2020. The Post-COVID-NE era was set between 1 August 2020 and 31 December 2021. Please refer to the text for detailed justification of the time periods used.

As anticipated based on the purpose of the UNOS heart allocation policy update, the overwhelming majority of candidates were transplanted as urgency status 2 from both cohorts (40.2% vs. 44.0%), followed by status 3 or 4 (data not shown). Average cold ischemic time was only 4 minutes longer in the Post-Cov group, yet the difference did reach statistical significance in our analysis (209 *±* 60 vs. 205 *±* 61 minutes; *p* = 0.006). Consistent with this finding is that the proportion of donor organs procured by a local OPO decreased from 29.6% to 22.1% in the Post-Cov era (*p* < 0.001). The incidence of PGD was very low overall in both of our study groups, with no significant difference between the two cohorts (0.5% vs. 0.3%; *p* = 0.356). Recipient 30-day survival exceeded 96% in both the Pre-Cov and Post-Cov cohorts with no statistically significant inter-group differences (log-rank *p* = 0.55; Figure 1).

## 4. Discussion

Our analysis highlights the complex and profound effects of the COVID-19 pandemic on the psychosocial health of the general population, which includes potential organ donors. Consistent with our hypothesis, we found a significant increase in the prevalence of SUD with an associated rise in drug intoxication-related mortality in our Post-Cov cohort. Fatal gunshot injuries were also more frequent in this group, likely associated, at least in part, with the increased homicide rate (8.7% vs. 7.5%; *p* = 0.036). Consistent with this finding, several papers have documented a clear increase in non-COVID-19 related deaths during the pandemic in the general population. According to the study by Lee and colleagues, the most significant increases were attributable to accidents and injuries followed by drug overdoses, and then assaults and homicides. A 20% increase in assaults and homicides was found over the study period [10]. Deaths related to drug overdose peaked in May 2020, while those due to accidents and assaults were at the highest in July 2020. Suicide rates were elevated throughout their study period [10].

The negative effects of substance abuse, such as alcohol, cocaine, methamphetamine, heroin, and, potentially, cannabis, on cardiac function are well documented in the literature [11,12,13,14,15]. Therefore, reviewing social history in detail is important when considering a donor organ for acceptance. While a low amount of ethanol consumption may potentially have a beneficial effect on cardiac health [11], chronic and excessive use can be deleterious [11,16]. The most commonly described cardiac abnormality associated with excessive alcohol intake is left ventricular dilation, progressing to manifest heart failure. This process is due to myofibroblast activation which promotes fibrosis [11]. Cocaine, on one hand, can provoke depressed myocardial contractility as it inhibits sodium/calcium exchange that results from decreased cellular sodium influx. At the same time, it acts as a stimulant by inhibiting central and peripheral catecholamine uptake. The cocaine-induced accumulation of catecholamines in the serum potentiates the activation of alpha and beta-adrenergic receptors [17]. This may provoke coronary vasospasm in sensitive individuals, prompting various degrees of cardiac dysfunction with wall motion abnormalities and secondary mitral regurgitation in the setting of focal or global myocardial ischemia. If persistent, infarction and fibrosis may ensue. In addition, myocardial metabolic demand rises owing to the augmented contractile force, and the overall risk of fatal arrhythmias increases. Methamphetamine exposure can affect cardiac function in the acute setting but also with chronic, long-term use. Acutely, it exerts a direct depressant effect on the myocardium that is potentially associated with protein damage and reduced adrenergic response [18]. Chronic methamphetamine use is a well-established cause of secondary cardiomyopathy. A meta-analysis by Manja and colleagues published in 2023 showed that methamphetamine-induced heart failure is increasing in prevalence and affects persons across all racial and socioeconomic groups [19]. Unfortunately, it is associated with significantly higher morbidity and symptom burden when compared to HF caused by other etiologies [19]. The effect of heroin abuse on myocardial function is less well described. One study of 85 patients showed right ventricular dysfunction in patients with chronic use [20].

Although illicit drug use is common among organ donors, the exact prevalence remains unknown at this time. This may be explained by the fact that cardiac echocardiogram is routinely performed for all potential organ donors to establish biventricular function, but heart grafts with repeatedly severe dysfunction may not be offered to recipients by the OPOs. Data from these excluded donors are not readily available. To ensure that there is no significant epicardial coronary artery disease among those with illicit drug use history, coronary angiogram is often requested by transplant centers prior to final organ acceptance. This ensures that only grafts with normal structure and function are utilized, which is critically important to maintain the best clinical outcomes. Transplant centers may also request additional studies prior to procurement, such as invasive hemodynamic evaluation to establish filling pressures and cardiac output, or inotrope challenge to gauge the potential improvement in myocardial contractility after transplantation.

Beyond representing a major epidemiological crisis, COVID-19 also induced widespread human psychological turmoil worldwide. Several studies have clearly demonstrated the tremendous toll of the pandemic on the mental health of the general population due to fear of contracting the disease, the adverse financial consequences in the setting of business closures and employee layoffs, mandated changes to the daily routine, travel restrictions, limitations on social gatherings, uncertainty related to misinformation, as well as the loss of close friends, family members, and loved ones [21,22,23,24,25,26]. In addition, it has been clearly demonstrated that survivors of COVID-19 infection are at significantly increased risk for developing a wide variety of psychiatric conditions, including post-traumatic stress disorder (PTSD) and depression [27,28]. These factors were compounded by the mandated, widespread, and prolonged social isolation imposed by governments worldwide aiming to eliminate, or at least contain, the virus. The combination of such circumstances may not only provoke increased anxiety, panic, and obsessive behavior but also worsen pre-existing depression and PTSD that may ultimately lead to increased alcohol and illicit substance use [29,30,31]. Indeed, several studies from the US and other counties have described this phenomenon during the pandemic with a disproportionate rise in fentanyl, heroin, and methamphetamine abuse. This is especially true in populations diagnosed with or at risk for SUD [32]. The consequent increase in the incidence of drug overdoses was evident by reviewing emergency department records [33]. These unfavorable trends in illicit drug use, anxiety, depression, and PTSD may potentially translate to increased violence and homicide rates, as we have demonstrated in our analysis.

Importantly, the shift in social history and mechanism of death had no impact on early PGD rates or recipient post-HT survival at 30 days in our study. Although we found that graft cold ischemic time was statistically longer in the Post-Cov cohort, the actual average difference was only 4 minutes versus the Pre-Cov group. This finding is critical to emphasize as cold ischemic time is one of the most important risk factors for the development of PGD. However, we believe that the relatively incremental increase we demonstrated is highly unlikely to be clinically significant and, therefore, to affect clinical outcomes. In addition, our data are consistent with the annual reports published by SRTR showing that transplant teams are travelling increasingly farther to procure organs in the updated heart allocation system without a detrimental effect on recipient or graft survival. Our findings are further corroborated by the fact that organ acceptance from outside of the local OPO donor service area has increased. Overall, this information is vital for the transplant community when reviewing donor characteristics, social history, and ultimately deciding on organ acceptance.

Given the ongoing, severe mismatch between the number of organs available for transplantation, especially the heart, and the number of potential recipients, achieving and maintaining superior post-transplant survival rates is critically important and is closely monitored by the relevant authorities. Graft quality, which is at least partially determined by the cause, mechanism, and manner of donor death, may have a profound impact on the incidence of PGD and peri-transplant outcomes. Therefore, a sudden, unfavorable shift in these variables, as provoked by the COVID-19 pandemic, may have a detrimental effect on recipient survival. Fortunately, our current analysis did not uncover such a negative impact with no difference in recipient survival at 30 days. However, ongoing scrutiny of post-transplant outcomes must remain a priority. Donor evaluation and graft acceptance practices may need to be adjusted based on updated research data in the future.

## 5. Conclusions

Our study confirms that pandemics, such as COVID-19, have a significant negative impact on the mental health and psychosocial life of the general population. Several studies highlight the increase in SUD and MHD after the latest pandemic [32]. This trend had multiple public health implications, including a significant increase in illicit substance use and fatal intoxication rates in organ donors. Despite this trend and, most importantly, these abrupt shifts in the mechanism and circumstance of donor death did not affect the rate of PGD and peri-operative mortality following heart transplantation. Ongoing monitoring and future studies are necessary to ensure that long-term outcomes also remain high and unaffected.

## Figures and Tables

**Table 1:** Selected donor characteristics in the Pre- and Post-COVID-19 national emergency (NE) declaration eras. Values presented are n (%) or mean ± SD. The Pre-COVID-NE era was defined as the period between 18 October 2018 and 12 March 2020. The Post-COVID-NE era was set between 1 August 2020 and 31 December 2021. Please refer to the text for detailed justification of the time periods selected for the purposes of this analysis. * donors had missing information on this variable in less than 4% of cases.

Variable	Pre-COVID-19 (n = 4915)	Post-COVID-19 NE (n = 5122)	*p*-Value
Mean donor age, years	29.9 ± 13.2	30.1 ± 12.6	0.54
Male, n (%)	3418 (69.5)	3602 (70.3)	0.41
**Donor race**			
African American, n (%)	797 (16.2)	935 (18.3)	0.026
White, n (%)	3944 (80.2)	4010 (78.3)	
Other, n (%)	174 (3.5)	177 (3.5)	
**Donor history**			
Active smoking, n (%)	517 (10.7)	534 (10.7)	0.98
Cocaine use, n (%)	1214 (25.1)	1165 (23.3)	0.036 *
Other illicit drug use, n (%)	2713 (55.8)	3033 (60.1)	<0.001 *
Heavy alcohol use, n (%)	767 (16.0)	839 (16.9)	0.228 *
**Donor cause of death**			
Anoxia, n (%)	2151 (43.8)	2282 (44.6)	0.335
Cerebrovascular/stroke, n (%)	630 (12.8)	618 (11.5)	
Head trauma, n (%)	1993 (40.5)	2109 (41.2)	
CNS tumor, n (%)	18 (0.4)	18 (0.3)	
Other, n (%)	123 (2.5)	106 (2.1)	
**Mechanism of death**			
Drug intoxication, n (%)	957 (19.5)	1194 (23.3)	<0.001
Gunshot wound, n (%)	740 (15.1)	865 (16.9)	0.01
Cardiovascular, n (%)	480 (9.8)	437 (8.5)	0.04
**Death circumstance**			
Homicide, n (%)	369 (7.5)	444 (8.7)	0.036
Suicide, n (%)	750 (15.1)	744 (14.5)	0.471
Primary graft dysfunction, n (%)	23 (0.5)	17 (0.3)	0.356
Cold ischemic time, minutes	205 ± 61	209 ± 60	0.006 *

## Data Availability

Although data used for this analysis are publicly available from SRTR, these will be provided upon reasonable request from Tamas Alexy, MD.
